# Calendar

**DOI:** 10.1111/iwj.14083

**Published:** 2023-01-30

## NORTH AMERICA

**26‐30 April 2023**

***Symposium on Advanced Wound Care (SAWC) Spring 2023***

**National Harbor, MD, USA**

www.sawcspring.com

**27‐28 April 2023**

***4th International Conference on Applied Microbiology and Infectious Diseases***

**Vancouver, Canada**

www.conferenceseries.com/microbiology-meetings

## EUROPE

**2 March 2023**

***15th Wound Congress ‐ CNC***

Hasselt, Belgium

www.wondzorg.net/nieuws/vijftiende-vlaams-wondzorgcongres

**18 April 2023**

WCS Wound Congress 2023

**Utrecht, The Netherlands**

www.wcs.nl/congres/wcs-wondcongres-2023

## INTERNATIONAL

**23‐24 February 2023**

***7*** ***th*** ***World Congress on Wound Healing and Critical Care***

**Dubai, United Arab Emirates**

www.aast.org

**25‐26 March 2023**

International Conference on Wound Healing Systems and Technologies (ICWHST)

**Tokyo, Japan**

www.waset.org

**17‐18 April 2023**

***International Conference on Wound Care***

**Seoul, Korea**

www.waset.org

